# Glances at the Hospitals

**Published:** 1900-11-17

**Authors:** 


### GLANCES AT THE HOSPITALS.

### THE SALOP INFIRMARY.

The cash receipts amounted to £5,400 and the expenditure to £5,898, there being on the date of the report a sum of £1,366 due to the treasurer. The income was derived as follows:—Subscriptions, £1,819; legacies, £1,511; collections, £948; dividends, £1,056; and miscellaneous, £83. The workmen's subscriptions are not given separately; but in turning over the subscription list it seemed to us that the sums received from this class were small in comparison to those received by other infirmaries. There were 1,033 in-patients, and of these 493 were discharged cured; 79 relieved; 368 made out-patients; 30 for other causes; and

62 died. The total number of out-patients was 18,302. A classified list of the diseases of the in-patients admitted during the year is given, but it merely gives the names of the diseases and the number of patients suffering from these diseases. No results are shown.

### THE LIVERPOOL HAHNEMANN HOSPITAL AND HOMŒOPATHIC DISPENSARIES.

THE receipts for the year amounted to £2,776 and the expenditure to £3,135, showing an adverse balance of £359. There were 447 in-patients treated during the year, and in addition to this number 161 were in the West Derby Convalescent Home. At the out-patient department the "attendances" reached the total 39,508, and at the Roscommon Street Dispensary the "attendances" were 20,724. A new operating theatre was opened in the hospital and equipped during the year. We did not notice any statement of the cost per bed occupied, nor of the weekly cost per head. The medical report of the in-patients treated during the year is carefully drawn up. The diseases are placed in groups, and separate columns show the results obtained in every disease. We learn that 59 per cent. were cured, 20 per cent. improved, 5·6 per cent. not improved, and 5·2 per cent. died. The table showing the operations gives like information to the general medical table. Of 134 operations there were 127 recoveries, 4 improved, 1 not improved, and 2 died. The anæsthetic used is not stated.

### THE CHESTERFIELD AND NORTH DERBYSHIRE HOSPITAL.

THE total income of the institution for 1899 was £1,944, and the total expenditure was £2,527, being £567 more than the income, the deficiency being met by an overdraft at the bank. A special appeal for funds has been made, but at the date of the report the result of the appeal could not be stated; but one donation of £500 from Mr. Edward Eastwood was announced. The average total cost per patient was £5 12s., and the average cost per day per head for diet was about 9½d. An analysis of the injuries and diseases of the in-patients for the year 1899 is given in tabular form, the number admitted, cured, relieved, died, and operated on being clearly stated. Altogether 419 injuries and diseases were under treatment, and of these 335 were cured, 20 were relieved, 24 died, 9 were unfit for or declined operation, and 31 remained in the hospital. 164 operations were performed. Of the 24 deaths 6 were due to burns and scalds, 3 to fractures of the pelvis, and 1 each to rupture of the bladder, tetanus, and septicæica.

### ST. BARTHOLOMEW'S HOSPITAL, CHATHAM.

THE total income for 1899 was £6,480, and the total expenditure amounted to £7,058; but £350 of the latter sum come under the head of extraordinary expenditure. It is proposed to build a new operating theatre, a lift from the receiving rooms to the level of the theatre, and a children's ward. All these are highly desirable additions; but it is to be hoped that they will not be commenced until the money is actually in hand, as paying off arrears is always a difficult thing for the managing committee of a hospital to accomplish, and at present the expenditure at Chatham exceeds the income. The medical report is the shortest and least instructive we have yet met. It contains nothing but some numerical statistics, and as to the diseases treated and the operations performed we hear absolutely nothing; and yet it would seem certain from the numbers under treatment that many interesting cases must have been in the hospital during the year 1899. On the last day of the year the hospital contained 64 in-patients, and there were 974 out-patients, 543 ophthalmic patients, and 307 casualties. The total nnmber of patients treated during the year was 16,402. Anæsthetics, nature not stated, were administered 398 times.

